# Inflammatory Status and Chronic Kidney Disease in Cats: Old and New Inflammatory Markers—A Pilot Prospective Study

**DOI:** 10.3390/ani13233674

**Published:** 2023-11-28

**Authors:** Annamaria Uva, Maria Alfonsa Cavalera, Oana Gusatoaia, Rossella Donghia, Floriana Gernone, Marco Silvestrino, Andrea Zatelli

**Affiliations:** 1 Department of Veterinary Medicine, University of Bari, 70010 Valenzano, Italy; annamaria.uva@uniba.it (A.U.); mariaalfonsa.cavalera@uniba.it (M.A.C.); oana.gusatoaia@uniba.it (O.G.); floriana.gernone@uniba.it (F.G.); marco.silvestrino@uniba.it (M.S.); 2 National Institute of Gastroenterology “S. de Bellis”, Research Hospital, 70013 Bari, Italy; rossydonghia@gmail.com

**Keywords:** feline, CKD, erythrocyte sedimentation rate, serum amyloid A, inflammation, APPs

## Abstract

**Simple Summary:**

Chronic kidney disease (CKD) is a major cause of morbidity and mortality in cats. Despite the increasing worldwide interest in feline CKD, the specific role of systemic inflammation in these patients remains poorly defined. The aim of the present prospective study was to assess serum amyloid A (SAA) and erythrocyte sedimentation rate (ESR) levels as markers of inflammation in cats diagnosed with CKD at IRIS stages 2–4. The results indicate a systemic inflammatory state associated with feline CKD, as both markers showed significantly higher levels in affected animals than in healthy ones. Compared to SAA, the rise in ESR appears to be more closely linked to advanced stages of the disease and could, therefore, correlate with the uremic condition.

**Abstract:**

This prospective study aimed to evaluate inflammatory status in cats affected by chronic kidney disease (CKD) at IRIS stages 2–4, using serum amyloid A (SAA) and the erythrocyte sedimentation rate (ESR) as inflammatory markers. Thirty-two cats with CKD and ten clinically healthy cats (i.e., control group) were enrolled. The recording of signalment data, complete physical examinations, and abdominal ultrasonography were performed for each animal. Additionally, ESR levels, complete blood count, clinical chemistry (including SAA determination), serum protein electrophoresis, and complete urinalysis were executed. This study’s results showed that mean ESR and SAA concentrations in cats with CKD were statistically higher compared to those of the control group (*p* = 0.0005 and *p* = 0.007, respectively). The SAA concentration was significantly increased at IRIS stages 2, 3, and 4 compared to the control group. Meanwhile, the ESR was significantly higher in cats at IRIS stages 3 and 4 (*p* = 0.0003 and *p* = 0.0007, respectively), but not at IRIS stage 2, compared to the control group. These results provide evidence that feline CKD is associated with a systemic inflammatory status. Moreover, the rise in ESR appears to be more linked to advanced stages of the disease and could, therefore, correlate with the uremic condition.

## 1. Introduction

Chronic kidney disease (CKD) is an irreversible process characterized by persistent (≥3 months) loss of function or structural changes in the kidneys, most often leading to a progressive decline in kidney function [1]. Feline CKD is the most common metabolic disease in cats and has been increasingly diagnosed over the past three decades, particularly in older cats. In 1990, the Purdue Veterinary Medical Database documented a renal failure prevalence of 1.6% in cats of all ages [2]. However, by 2000, this prevalence had increased to a higher value of 9.6% [3]. Age is recognized as a significant risk factor influencing CKD in cats [2,4,5,6], with prevalence rising to 42% in senior-adult cats and 81% in geriatric cats [7]. Indeed, CKD represents the primary cause of mortality in cats over 5 years of age [8]. Due to its significant clinical relevance, interest in feline CKD is increasing worldwide, and the International Renal Interest Society (IRIS) recommends early detection, disease staging, and initiation of standard therapy to slow the progression of this potentially life-threatening disease [9].

In human medicine, 30–75% of CKD patients show systemic chronic inflammation, which is linked to disease progression [10,11,12,13,14] and results from various processes, including increased pro-inflammatory cytokines, metabolic acidosis, and uremia-induced oxidative stress [15,16,17,18,19]. Elevated levels of inflammatory markers (e.g., C-reactive protein [CRP], interleukin (IL)-6, and tumor necrosis factor-α (TNF-α)) are commonly observed [11,12,20,21,22], along with an increased erythrocyte sedimentation rate (ESR), in CKD patients, as reported in several studies [23,24,25,26,27,28,29]. In human hematology, ESR is a commonly performed laboratory test that measures the distance that red blood cells travel in a tube of anticoagulated blood in a given unit of time, usually an hour. This determination is usually used as an index of systemic inflammation in infectious and non-infectious diseases, albeit in a manner lacking specificity. The level of ESR is influenced by acute phase proteins (APPs), mainly fibrinogen, which reduce the negative charge between erythrocytes, leading to the faster aggregation of erythrocytes and an increase in ESR [30].

The role of systemic inflammation in feline CKD is not as firmly established as it is in human patients affected by CKD. A few previous studies mentioned renal disease as a cause of increased serum amyloid A (SAA) [31,32], which is the major positive APP in cats [33,34]. In addition, a further study reported higher SAA and hepcidin levels in cats with CKD compared to the healthy population [32]. While the ESR is extensively used as an inflammatory marker in human medicine, its use in veterinary medicine is still limited. Although studies of dogs have recently highlighted the valuable and timely use of ESR as an inflammatory biomarker for conditions such as osteoarthritis, babesiosis, ehrlichiosis, and active leishmaniosis [35,36,37,38], there are currently limited data available on ESR in sick cats, including those with CKD [39,40]. To the best of our knowledge, the only information available on this marker in cats mainly concerns the effects of time, temperature, and length of storage, as well as packed cell volume, on the ESR value. Furthermore, as the ESR measurement was carried out in cats using various techniques (i.e., the Wintrobe method, the Capillary method, and using an automated device) [39,40], drawing meaningful comparisons between the data is challenging.

This prospective study aims to complete the following steps: (i) evaluate ESR and SAA in cats with CKD according to IRIS staging; (ii) compare ESR and SAA between healthy and CKD cats; and (iii) assess the existence of a correlation between ESR and SAA concentration in CKD cats.

## 2. Materials and Methods

### 2.1. Study Population and Data Collection

From January 2022 to June 2023, client-owned cats older than two years and of any sex, weight, breed, and reproductive status referred to the Veterinary Teaching Hospital of the University of Bari “Aldo Moro” (Bari, Italy) were prospectively recruited if affected by a stable CKD classified as being at IRIS stages 2 to 4 [9] (i.e., CKD group). In this regard, CKD diagnosis had to be based on the presence of at least two of the following symptoms: (i) renal structural abnormalities; and/or (ii) “inappropriately diluted” urine (i.e., urine-specific gravity [USG] < 1.035 in a dehydrated animal; [9]) without another identifiable cause of polyuria, namely renal disease; and/or (iii) increased serum creatinine concentration without an identifiable cause of pre-renal or post-renal azotemia; and/or (iv) proteinuria of renal origin confirmed based on a repeated urine-to-protein ratio (UPC) > 0.2 and inactive sediment [41]. CKD was considered stable if blood creatinine had not changed by more than 15% in the previous 2–4 weeks after the enrolment. After diagnosis, CKD was staged according to IRIS guidelines [9]. This allowed the enrolment of cats with chronic renal failure (i.e., IRIS stages 2–4), wherein an increase in uremic toxins, not present at IRIS Stage 1, could potentially act as a pro-inflammatory stimulus.

Moreover, shelter and client-owned cats considered clinically healthy based on comprehensive clinical, hematological, biochemical, and urine evaluations performed within pre-adoption and annual screenings, respectively, were also enrolled as a control group.

For each animal enrolled, signalment data (i.e., breed, age, and sex), baseline information (i.e., clinical history, concomitant treatments, and previous information on laboratory tests), and complete physical examination findings were recorded. An abdominal ultrasound examination (US) was carried out to assess renal imaging and identify any anatomical abnormalities and/or structural changes. Furthermore, the measurement of the ESR level, complete blood count (CBC), clinical chemistry, serum proteins electrophoresis, and complete urinalysis (including USG measurement, urine dipstick and sediment evaluation, and UPC measurement) were performed. Urine culture was also executed in the cases of positive urinary sediment recorded in animals with lower urinary tract signs in order to identify cats affected by urinary tract infections (UTIs) [42].

Cats with a confirmed diagnosis of acute kidney injury within the previous 28 days and/or azotemia of pre-renal or post-renal origin were excluded. Cats with suspected or confirmed infectious diseases (e.g., UTIs, feline infectious peritonitis, and retrovirus infections) and/or illnesses (e.g., neoplastic, diabetes mellitus, and hyperthyroidism), as well as those being treated with drugs able to influence the immune response and inflammatory markers (anti-inflammatory and/or immunosuppressive drugs), were also excluded.

### 2.2. Sample Collection

Hematological and biochemical analyses were carried out on peripheral blood samples obtained via jugular venipuncture. For CBC and ESR measurement, a 2 mL blood sample was collected in a BD Vacutainer^TM^ K3-EDTA tube. Serum samples were obtained via the centrifugation (15 min at 1500× *g*) of 5 mL blood collected in a BD Vacutainer^®^ clot activator serum tube. Blood and serum samples were stored at 4–8 °C and processed within 12 h of collection. All urine specimens were sampled via ultrasound-guided cystocentesis and stored in BD Vacutainer^®^ urinalysis preservative tubes (5 mL) for physical–chemical examination. Urine samples were stored at room temperature and examined within 4 h of collection.

### 2.3. Laboratory Tests

The results of CBC (Siemens, ADVIA 2120, Erlangen, Germany), serum biochemical analysis (Beckman Coulter, Clinical Chemistry Analyzer AU680, Indianapolis, IN, USA), and serum protein electrophoresis (SEBIA Italia S.r.l., Capillarys 2 Flex Piercing, Florence, Italy) were acquired via the same methods in all tested samples. Microscopic blood smear examination was also performed for all samples. Cats were considered anemic based on hematocrit and hemoglobin values. The anemia was staged as mild, moderate, or severe according to the method of Tvedten [43]. The regenerative nature of anemia was assessed via the evaluation of the red blood cell morphology at the blood smear (e.g., microcytosis and hypochromia).

The ESR measurement was performed within one hour of collection using an automated point-of-care device (MINI-PET, DIESSE, Diagnostica Senese S.p.A., Siena, Italy). Serum amyloid A concentrations were measured using a turbidimetric immunoassay (LZ-SAA Standard Q; product code: G-SZ75; Eiken Chemical Co., LTD., Tokyo, Japan), performed via a chemistry analyzer (AU 5800; Beckman Coulter Inc., Brea, CA, USA) [44,45] with an RI of 0.1–0.5 µg/mL. USG was measured using a refractometer (Leica Vet 360, Misco Products Division, Cleveland, OH, USA). Urine dipstick examination (including pH, glucose, ketone bodies and bilirubin) was interpreted as recommended by the manufacturer (Combur 9 Test, Roche, Rotkreuz, Switzerland). In order to determine the UPC ratio, the protein concentration (mg/dL) was assessed using pyrogallol red-molybdate assay, while the serum creatinine (mg/dL) was measured through the Jaffé method in undiluted urine. Urine sediment was obtained via the centrifugation (10 min at 900× *g*) of 5 mL of urine, followed by the removal of 4.5 mL of supernatant, and the resuspension of the remaining 0.5 mL of urine. A sample of 12 μL of the resuspended urine was microscopically assessed. Red and white blood cells were expressed as the mean number of cells/high power field (hpf; 40× magnification). Urine sediment with bacteriuria and/or >5 red or white blood cells/hpf was considered to be indicative of an active sediment.

### 2.4. Statistical Analysis

The normal distribution of the results was checked via the Kolmogorov–Smirnov test. Our results were reported as the mean and standard deviation (M ± SD) or the median and interquartile range if normally or non-normally distributed, respectively, as well as as frequencies and percentages (%) for categorical variables. For testing the associations between independent groups (CKD Group vs. Control Group), a Wilcoxon Rank Mann–Whitney for continuous variables and Fisher’s exact test for categorical variables were used, while the Kruskal–Wallis equality rank test was used to compare more than two independent groups. Dunn’s test was performed for multiple pairwise comparisons. The Spearman rank correlation coefficient was used to test the strength and direction of the association between the two variables examined (i.e., ESR or SAA between blood parameters examined). The strength of this relationship, according to the correlation coefficient value (ρ), was qualified as follows: weak for rs = 0 to 0.3; moderate for rs = 0.3 to 0.6; strong for rs = 0.6 to 0.9; and very strong for rs = 0.9 to 1. The results were presented as a rho correlation and *p*-value (in brackets). When testing the null hypothesis of no association, the probability level of error at two tails was set at 0.05. All the statistical computations were performed using StataCorp. (College Station, TX, USA) 2023. Stata Statistical Software: Release 18. College Station, TX: StataCorp. LLC.

## 3. Results

### 3.1. Study Groups

During the study period, sixty-two cats were initially evaluated. In total, 20 cats were excluded, including 10 cats from the CKD group (i.e., *n* = 6 in IRIS stage 1; *n* = 3 incomplete data; *n* = 1 concomitant lower urinary tract disease) and 10 apparently clinically healthy cats, because of the presence of laboratory abnormalities (i.e., *n* = 7 leucocytosis, *n* = 1 mild anemia, *n* = 1 elevated SAA levels, and *n* = 1 lymphocytosis). Ultimately, 42 cats were enrolled in this study, including 32 animals in the CKD group and 10 in the control group.

In the CKD group (9 ± 3.74 years), 23 out of 32 cats were domestic shorthair, and 9 were other breeds (*n* = 3 British Shorthair, *n* = 3 mixed Persian, *n* = 2 Nebelung, *n* = 1 domestic longhair). Moreover, 10 cats were spayed females, 2 were intact females, 19 were castrated males, and 1 was an intact male.

In the control group (2.25 ± 2.33 years), all cats were domestic shorthair, including four spayed, five intact females, and one intact male. Cats in the CKD group were significantly older than those in the control group (*p* = 0.01).

In the CKD group, 11 (34.4%), 13 (40.6%), and 8 (25%) out of 32 cats were at IRIS stages 2, 3, and 4, respectively.

### 3.2. Laboratory Analysis

#### 3.2.1. Comparison between Control and CKD Groups

The main parameters of interest for the study have been compared between the CKD and control groups (Table 1). In total, 13 out of 32 cats in the CKD group had non-regenerative anemia classified as mild in *n* = 9 and moderate in *n* = 3 animals. Mild non-regenerative anemia was identified in 2/11 (18%), 5/13 (38%), and 2/8 (25%) cats at IRIS stages 2, 3, and 4, respectively. Moderate non-regenerative anemia was identified in 1/11 (9%), 1/13 (8%), and 1/8 (12.5%) cats at IRIS stages 2, 3, and 4, respectively.

**Figure 1:**
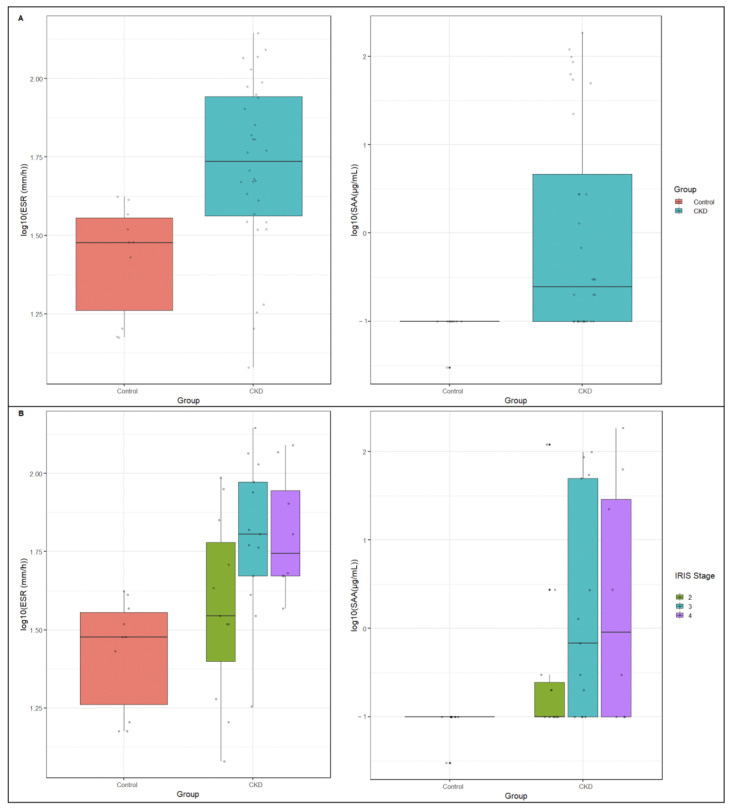
Box plots of ESR and SAA levels in the control and CKD groups (**A**), as well as according to the IRIS staging (**B**).

Mean ESR and SAA concentrations in CKD cats were significantly higher compared to the control group (Table 1). ESR and SAA levels in cats with CKD according to the IRIS stages and compared to healthy cats are shown in Table 2 and Figure 1.

#### 3.2.2. Evaluation of ESR in CKD Cats

The ESR was significantly higher in IRIS 3 and 4 cats (*p* = 0.0003, *p* = 0.0007, respectively) compared to the control group, while no difference was found between IRIS 2 cats and the control group (Table 2). Among cats with CKD, the ESR was significantly elevated in IRIS 3 and 4 compared to IRIS 2, while there was no significant difference between IRIS 3 and 4 cats (Table 2). In the CKD group, the ESR showed a moderate negative correlation with hematocrit value, hemoglobin, and albumin concentration, while it exhibited a moderate positive correlation with urea concentration and UPC (Table 3).

#### 3.2.3. Evaluation of SAA in CKD Cats

The SAA concentration was significantly higher in CKD cats at IRIS stages 2, 3, and 4 compared to the control group. However, among cats with CKD, there were no significant differences in SAA concentrations between IRIS stages 2, 3, and 4 (Table 2). In the entire CKD population, SAA showed a moderate negative correlation with hematocrit value, hemoglobin concentration, and albumin concentration, while it exhibited a moderate positive correlation with urea concentration, alpha-2-globulins, and UPC (Table 4).

## 4. Discussion

The present study provides, for the first-time, data regarding ESR in cats affected by CKD, being significantly higher compared to the value detected in healthy animals. Moreover, the CKD group exhibited increased levels of SAA compared to the control group, supporting earlier findings [31,32].

Consistent with human studies, cats at advanced stages of CKD (IRIS stages 3 and 4) exhibited a significantly higher ESR level compared to the control group. Conversely, for cats affected by CKD IRIS stage 2, no significant difference in ESR compared to the control group was found. These findings suggest that advanced CKD is associated with a systemic inflammatory status, likely related to uremia, as we observed a moderate positive correlation between ESR and urea levels. Indeed, at advanced CKD stages, the accumulation of uremic toxins promotes the production of carbonyl compounds [15], which exacerbate oxidative stress, a well-known, strongly pro-inflammatory condition [16].

In the CKD group, significant moderate negative correlations between ESR and both hematocrit value and hemoglobin concentration were found. In human medicine, existing evidence indicates that non-inflammatory conditions influencing ESR are primarily associated with hematocrit value, size, shape and number of red blood cells, and blood interferents, such as lipemia and hemolysis [46,47]. Scant data are available for cats regarding the influence of hematocrit value on ESR [40], while no evidence is present for blood interferents. However, in this regard, none of the enrolled cats had hemolytic or lipemic samples. Conversely, considering that 13 out of 32 CKD cats were anemic, it is not possible to exclude the possibility that the increase in ESR in cats with advanced CKD may also partly be a consequence of their anemic state.

In the present study, a significant increase in the mean SAA concentration in the CKD group compared to the control group was found, as previously reported in [32]. The increase in the SAA level in the context of a chronic disease such as CKD may be justified, considering that changes in APPs concentration have also been observed during chronic inflammatory states [48,49]. The increase in SAA herein reported at CKD IRIS stages 2–4 suggests its potential use as a marker of inflammation in this disease. Furthermore, the existence of a moderate positive correlation between SAA concentration and urea levels (as also noted for ESR) suggests a potential association between more severe inflammation and advanced CKD.

Similar to ESR, statistically significant moderate negative correlations between SAA and both hematocrit and hemoglobin concentrations were observed within the CKD group. Although a correlation between hematocrit/hemoglobin and SAA is not sufficient to establish a cause-and-effect relationship between anemia and inflammation, previous evidence indicates that inflammatory disease is a well-known cause of anemia in cats [50], including those with CKD [32]. However, it cannot be excluded that individual parameters (i.e., hematocrit, hemoglobin, SAA) move in parallel due to other factors.

In the CKD group, both ESR and SAA values exhibited a moderate negative correlation with albumin concentration. This was not unexpected since albumin levels tend to decrease during inflammation, as it is a negative APP [48].

No correlation between ESR and globulin fractions in serum protein electrophoresis was found. In contrast, a correlation was observed between SAA and α2 globulins, consistent with the expected result, given that many of the APPs moved within the α globulin fraction of the electrophoretic pattern [51].

Within the CKD cohort, a moderate positive correlation was found between proteinuria and both ESR and SAA. In feline chronic kidney disease, proteinuria serves as a negative prognostic factor [52]. It could be interesting to explore the cause-and-effect relationship between proteinuria and inflammation. In cats, proteinuria is acknowledged to contribute to the progression of renal damage by instigating interstitial inflammation and fibrosis around renal tubules [53]. On the other hand, in humans, inflammation has been identified as a risk factor for proteinuria [54]. In this context, the management of inflammation may emerge as a pivotal therapeutic strategy for felines experiencing proteinuric nephropathy.

Interestingly, despite the higher levels of both ESR and SAA in cats with CKD when compared to healthy cats, no correlation between them was found. The absence of a correlation between ESR and SAA in cats with chronic kidney disease is likely due to the fact that these two laboratory parameters reflect distinct inflammation-related processes. Although both of these markers are elevated in cats with kidney disease, their underlying mechanisms of increase may not be closely interconnected. Moreover, it is possible that SAA demonstrates higher sensitivity among the entire population of cats with renal disease compared to ESR. This suggests that SAA may exhibit a more responsive and sensitive response to inflammatory conditions related to kidney disease, while ESR could only increase in advanced stages of kidney disease or in response to specific additional conditions.

The increased ESR and SAA levels noted in cats with CKD provide evidence that feline CKD, like human CKD, is associated with a systemic inflammatory status. This finding is quite relevant, since, in both cats and humans, chronic inflammation and oxidative stress play a critical role in the progression of kidney damage [55,56]. In chronic inflammation, as mentioned above, an abnormal and prolonged acute phase reaction (APR) triggered by ongoing stimuli (e.g., uremia, anemia) can promote the underlying tissue damage associated with the disease and lead to additional complications [29,57]. In the current context, a growing body of evidence supports considering inflammation and its impact on CKD progression as a plausible therapeutic target in the clinical management of cats affected by CKD. Combining SAA and ESR, as well as other inflammatory markers, as diagnostic and follow-up tools for inflammation has potential value, especially if therapeutic interventions targeting chronic inflammation can be integrated into the comprehensive CKD treatment strategy.

This study has a number of limitations, such as the limited number of enrolled cats in the control group, due to the strict inclusion criteria. The significant difference in the age between control and CKD groups could represent an additional limitation since increases in SAA and ESR levels have been described in old cats [34] and elderly human patients [58], respectively. However, though it might have been expected that the CKD group would include elderly cats [2,4,5,6], the enrolment of an age-matched control group is challenging in clinical practice.

In our study, the evaluation of the inflammatory response was investigated using SAA and ESR as markers. Nevertheless, it is worth noting that measuring other APPs in cats, such as alpha-1-glycoprotein and hepcidin, could have provided additional and complementary insights. Additional research is necessary to thoroughly investigate this issue. Finally, the measurement of ESR in cats currently has a number of limitations, including the lack of a validated method in the scientific literature for the point-of-care instrument used in the present study, as well as a species-specific RI for the cats; however, the statistical analysis herein included a comparison of the population of cats with CKD to the control group of clinically healthy cats, thus mitigating the potential effect of the lack of a validated reference interval. In addition, unlike human medicine, correction factors for ESR based on hematocrit values in cats are still not available, potentially impairing the evaluation of this parameter in animals with anemia.

## 5. Conclusions

In conclusion, this study shows that feline CKD is associated with a systemic inflammatory status characterized by increased levels of ESR and SAA, a major acute-phase protein. The rise in ESR appears to be more closely linked to advanced stages of the disease and could, therefore, correlates with the uremic condition. Further studies are needed to better understand if the regular assessment of inflammatory markers (i.e., SAA and ESR) in cats affected by CKD can provide a deeper understanding of the disease’s severity and the treatment’s effectiveness.

## Figures and Tables

**Table 1:** Comparison of the parameters of interest for this study between the control and CKD groups and the number (percentage) of animals in the reference interval for each parameter (*n* = 42).

Parameter (Unit) * Reference Interval *	Total Cohort (*n =* 42)	Group		*p* ^^^
		Control (*n =* 10)	CKD (*n =* 32)	
ESR (mm/h)	45.00 (33.00–71.00)	30.00 (16.00–37.00)	54.50 (36.00–88.00)	0.0005
SAA (µg/mL)	0.10 (0.10–2.73)	0.10 (0.10–0.10)	0.25 (0.10–12.47)	0.0007
0.1–0.5	28 (66.67)	9 (90.00)	19 (59.38)	0.12 ^Ψ^
HCT (%)	32.25 (25.30–36.10)	34.25 (33.20–39.00)	29.90 (24.65–35.90)	0.02
28–43	30 (71.42)	9 (90.00)	21 (65.62)	0.47 ^Ψ^
Hg (g/dL)	10.20 (8.00–11.90)	11.35 (9.80–12.10)	9.95 (7.68–11.45)	0.08
9.5–15	28 (66.67)	9 (90.00)	19 (59.38)	0.12 ^Ψ^
Urea (mg/dL)	94.00 (57.00–178.00)	51.00 (45.00–57.00)	134.50 (71.00–258.00)	<0.0001
29–60	12 (28.57)	9 (90.00)	3 (9.38)	<0.001 ^Ψ^
Creatinine (mg/dL)	2.59 (1.75–4.53)	1.12 (0.95–1.19)	3.88 (2.12–5.28)	<0.0001
0.9–1.6	10 (23.8)	10 (100.00)	0 (0.00)	<0.001 ^Ψ^
Albumin (g/dL)	3.25 (2.92–3.79)	3.77 (3.25–3.79)	3.15 (2.85–3.80)	0.20
Alpha-1 Globulins (g/dL)	0.08 (0.07–0.10)	0.08 (0.06–0.09)	0.09 (0.07–0.11)	0.10
Alpha-2 Globulins (g/dL)	1.17 (0.98–1.33)	1.11 (0.95–1.16)	1.20 (1.02–1.37)	0.11
Beta Globulins (g/dL)	0.86 (0.79–1.09)	0.08 (0.72–0.85)	0.93 (0.80–1.14)	0.005
Gamma Globulins (g/dL)	1.39 (1.16–1.98)	1.32 (1.12–1.64)	1.65 (1.23–2.01)	0.23
USG	1.021 (1.017–1.046)	1.054 (1.046–1.066)	1.019 (1.015–1.023)	<0.0001
UPC	0.29 (0.14–0.56)	0.16 (0.12–0.27)	0.38 (0.15–1.67)	0.03

* As median and interquartile range (IQR) for continuous variables and percentage (%) for categorical variables. ^ Wilcoxon rank-sum test (Mann–Whitney), ^ψ^ Fisher’s test. Abbreviations: HCT, hematocrit; Hg, hemoglobin; ESR, erythrocyte sedimentation rate; SAA, serum amyloid A; USG, urine-specific gravity; UPC, urinary protein-to-creatinine ratio.

**Table 2:** Comparison between ESR and SAA between the control and CKD groups according to the IRIS stages.

Parameters *	Control (*n =* 10) (*a*)	IRIS Stages			*p* ^^^	*b* vs. *a* ^Ψ^	*c* vs. *a* ^Ψ^	*d* vs. *a* ^Ψ^	*c* vs. *b* ^Ψ^	*d* vs. *b* ^Ψ^	*d* vs. *c* ^Ψ^
		2 (*n =* 11) (*b*)	3 (*n =* 13) (*c*)	4 (*n =* 8) (*d*)							
ESR	30.00 (16.00–37.00)	35.00 (19.00–71.00)	64.00 (47.00–94.00)	56.00 (47.00–98.50)	0.001	0.07	0.0003	0.0007	0.02	0.03	0.45
SAA	0.10 (0.10–0.10)	0.10 (0.10–0.30)	0.68 (0.10–49.84)	1.52 (0.10–42.60)	0.006	0.03	0.0006	0.002	0.09	0.12	0.50

* As median and interquartile range (IQR) for continuous variables and percentage (%) for categorical variables. ^ Kruskal–Wallis rank test; ^ψ^ Dunn’s test of Multiple Comparisons. Abbreviations: ESR, erythrocyte sedimentation rate; SAA, serum amyloid A.

**Table 3:** Correlation matrix between ESR and other recorded parameters.

Parameters	Total Cohort ρ (*p*-Value)	Control ρ (*p*-Value)	CKD ρ (*p*-Value)
SAA	0.45 (0.003)	0.47 (0.18)	0.24 (0.18)
HCT	−0.55 (0.0002)	0.13 (0.71)	−0.56 (0.001)
Hg	−0.51 (0.0006)	0.30 (0.39)	−0.57 (0.0008)
Urea	0.58 (0.0001)	0.26 (0.46)	0.38 (0.03)
Creatinine	0.60 (<0.0001)	0.34 (0.33)	0.35 (0.05)
STP	−0.15 (0.41)	0.10 (0.77)	−0.15 (0.41)
Albumin	−0.46 (0.003)	0.30 (0.39)	−0.53 (0.003)
Alpha-1 Globulins	0.03 (0.86)	−0.35 (0.31)	−0.13 (0.49)
Alpha-2 Globulins	0.12 (0.45)	−0.06 (0.86)	−0.03 (0.86)
Beta Globulins	0.39 (0.01)	−0.47 (0.16)	0.20 (0.29)
Gamma Globulins	0.22 (0.17)	0.03 (0.93)	0.13 (0.47)
UPC	0.39 (0.03)	−0.11 (0.76)	0.39 (0.03)

ρ, Spearman’s rho. Abbreviations: ESR, erythrocyte sedimentation rate; SAA, serum amyloid A; HCT, hematocrit; Hg, hemoglobin; STP, serum total protein; USG, urine-specific gravity; UPC, urinary protein-to-creatinine ratio.

**Table 4:** Correlation matrix between SAA and other recorded parameters.

Parameters	Total Cohort ρ (*p*-Value)	Control ρ (*p*-Value)	CKD ρ (*p*-Value)
ESR	0.45 (0.003)	0.47 (0.18)	0.24 (0.18)
HCT	−0.44 (0.003)	−0.29 (0.50)	−0.35 (0.05)
Hg	−0.45 (0.003)	−0.06 (0.90)	−0.43 (0.01)
Urea	0.59 (0.0001)	−0.29 (0.50)	0.45 (0.01)
Creatinine	0.55 (0.0002)	−0.12 (0.80)	0.32 (0.07)
STP	0.09 (0.55)	−0.12 (0.80)	0.07 (0.68)
Albumin	−0.50 (0.001)	−0.06 (0.90)	−0.52 (0.004)
Alpha-1 Globulins	0.20 (0.21)	−0.36 (0.42)	0.14 (0.44)
Alpha-2 Globulins	0.52 (0.0007)	−0.17 (0.70)	0.57 (0.001)
Beta Globulins	0.44 (0.005)	−0.52 (0.09)	0.38 (0.04)
Gamma Globulins	0.19 (0.24)	−0.17 (0.70)	0.14 (0.47)
UPC	0.49 (0.001)	−0.06 (0.90)	0.46 (0.009)

ρ, Spearman’s rho. Abbreviations: SAA, serum amyloid A; ESR, erythrocyte sedimentation rate; HCT, hematocrit; Hg, hemoglobin; STP, serum total protein; USG, urine-specific gravity; UPC, urinary protein-to-creatinine ratio.

## Data Availability

The data presented in this study are available on request from the corresponding authors.
